# Association between physical activity, sedentary time, participation in organized activities, social support, sleep problems and mental distress among adults in Southern Norway: a cross-sectional study among 28,047 adults from the general population

**DOI:** 10.1186/s12889-022-12769-x

**Published:** 2022-02-23

**Authors:** Tonje Holte Stea, Susanne Aune Solaas, Annette Løvheim Kleppang

**Affiliations:** 1 Department of Health and Nursing Science, University of Agder, Postbox 422 4604, Kristiansand, Norway; 2 Department of Child and Adolescence Mental Health, Sørlandet Hospital, Kristiansand, Norway

**Keywords:** Symptoms of anxiety and depression, Mental distress, Physical activity, Sedentary time, Organized activities, Social support, Sleep

## Abstract

**Abstract:**

**Background:**

Identification of modifiable factors associated with poor mental health is crucial to develop targeted and effective intervention strategies for prevention of mental distress and illness in the general population. Thus, the aim of the present study was to examine the association between low level of leisure-time physical activity, high sedentary time, low participation in organized activities, low social support, sleep problems, and mental distress in a large sample of Norwegian adults.

**Methods:**

A cross-sectional study was completed by 28,047 adults (≥18 years old) in southern Norway by filling out an online self-report questionnaire. Multivariable binary logistic regression models, stratified according to gender and adjusted for age and perceived financial situation, were used to examine possible associations between unhealthy lifestyle behaviors, participation in organized activities, social support, and mental distress.

**Results:**

Our results showed an increased odds of having mental distress among men reporting low leisure-time physical activity (OR: 1.18; 95%CI: 1.03–1.37), high sedentary time (1.32; 1.16–1.51), low involvement in organized activities (1.43; 1.25–1.64), low social support (2.55; 2.18–2.99), and sleep problems (7.29; 6.35–8.37) compared to the rest of the male population. For women, the results showed increased odds of mental distress among those reporting high sedentary time (1.25; 1.11–1.39), low involvement in organized activities (1.60; 1.42–1.80), low social support (2.71; 2.39–3.06), and sleep problems (5.78; 5.15–6.50) compared to the rest of the female population. For both men and women, results also indicated that mental distress was increased among younger adults and among those reporting financial difficulties compared to the rest of the population.

**Conclusion:**

Our results showed an association between unhealthy lifestyle behaviors, low participation in organized activities, low social support and mental distress, and that the strength of the association varied substantially. These findings provide increased knowledge about the relationship between modifiable lifestyle factors and mental health which should have implications for future public health efforts.

## Introduction

Mental disorders are among the leading causes of disability and disease worldwide and estimates suggest a higher burden of mortality among individuals and populations with mental disorders [1, 2]. Across the European Union, mental illnesses have been reported to affect more than one in six adults, and the social and economic costs are substantial [3]. The strong positive relationship between mental distress and mental disorder and the strong negative relationship between mental distress and mental wellbeing [4], underlines the importance of identifying modifiable factors that may prevent development of mental distress and mental illness in the general population.

Healthy lifestyle behaviors, such as higher levels of physical activity, have been associated with reduced risk of mental distress [5]. Moreover, evidence reported by research literature reviews suggest a dose-response relationship and that any level of physical activity seem to decrease the risk of developing depressive symptoms [6, 7]. Observations among a representative sample of Canadian adults have confirmed a dose-response relationship between objectively measured physical activity and improved mental health but also suggested that physical activity and mental health associations could be hampered by daily sedentary time [8]. A positive association between sedentary time and mental distress, independent of physical activity level was also confirmed by a cross-sectional study among Belgian adults [9]. In addition, studies have identified short sleep duration as a risk factor for depression [10, 11] and mental distress [12], and that sleep distrubances are independently and strongly associated with poor health-related quality of life in middle-aged and older adults [13].

Involvement in organized activities, such as cultural engagement and participation in sport, has also been suggested to be an independent risk-reducing factor for development of depressive symptoms [14, 15]. Further, as involvement in social organized activities seemed to predict both higher quality of life and lower levels of depressive symptoms [16], development and maintenance of organized sport and recreational activities that are socially and culturally relevant has been supported [17].

Several studies, especially among elderly, have also confirmed that social support plays an independent and important role in enhancing and maintaining improved mental wellbeing [18–21]. Further, results indicating a significant association between social support from friends, spouse or relatives and reduced odds of panic disorder and mental distress, especially when the severity of mental health problems are low, highlights the importance of social networks and support [22]. On the other hand, a cross-sectional register study among Danish adults showed that individuals with less frequent social encounters than desired and those with less contact with family and friends than once a month had increased risk for poor mental health [23].

Most previous studies investigating associations between modifiable lifestyle behaviors, participation in organized activities, social support and mental distress are hampered by low participation rate, low quality of the studies and focus on few explanatory factors of mental distress [5, 24, 25]. Thus, the present study contributes to the existing literature by examining possible associations between a range of modifiable factors, including low level of leisure-time physical activity, high sedentary time, low participation in organized activities, low social support, sleep problems and mental distress among a large sample of Norwegian adults.

## Methods

### Participants and procedures

The present cross-sectional study was part of the Norwegian Counties Public Health Surveys (NCPHSs), designed to collect information about health-related behaviors, health, well-being, and quality of life among the adult population across Norway [26]. For inclusion in the present study, a random sample of 75,191 adults (≥18 years old) from all 30 municipalities in southern Norway was drawn from the National Register (31.6% of the adult population in this region). Of these, a total of 10,862 had actively reserved against participation in surveys in the Contact and Reservation Register and was removed from the sample. After further removal of deceased individuals, those registered with unverified contact information, or address outside the included municipalities, a total of 61,611 residents were invited by SMS and e-mail to participate in the present study. Written and oral information about the study was provided to invited participants and through official webpages and social media. Six random participants each received a gift card worth 4000 NOK (approximately 380 EUR). A total of 28,047 adults agreed to participate (response rate, 45.5%). All participants gave their consent by filling out an online consent form and proceeded by filling out an online self-report questionnaire between September and October 2019. Approximately 15 min were used to complete the study.

Participation in the present study was voluntary, and all participants had the opportunity to withdraw from the study at any time. The National Institute of Public Health was responsible for collecting and anonymizing data. Independent researchers who did not participate in the data collection process or had access to personally identifiable information analyzed the data. The Norwegian Institute of Public Health holds legal responsibility for the public health survey, and the study was conducted in line with the Declaration of Helsinki. Ethical approval and research clearance were obtained from the ethical committee at the University of Agder.

### Measures

All questions, response alternatives, and variable definitions used in the study are presented in Table 1.

**Table 1:** Questions, response alternatives, and variable definitions

Questions	Response alternatives (coded)	Variable definitions
***Mental distress***		
During the past week, to what degree have you been bothered with nervousness or inner concern?	Not bothered (1), a little bothered (2), somewhat bothered (3), extremely bothered (4)	No mental distress (reference) Mental distress (> 2.0)
During the past week, to what degree have you been bothered with fear or anxiousness?		
During the past week, to what degree have you been bothered with hopelessness about the future?		
During the past week, to what degree have you been bothered with unhappiness or sadness?		
During the past week, to what degree have you been bothered with concern or restlessness?		
***Leisure-time physical activity***		
How often do you usually exercise? (average)	Never (0), <once a week (0.5), once a week (1), 2–3 times/week (2, 5), 4–5 times/week (4.5), approximately every day (6.5)	Adherence to physical activity recommendations (75 min/week or more with high intensity or 150 min/week or more with moderate intensity) (reference) Not adhering to physical activity recommendations
For how long do you usually exercise each time? (average)	< 15 min (8), 15–29 min (22), 30–60 min (45), > 60 min (75)	
How hard do you usually exercise? (average)	Easy without being short of breath or sweaty (low intensity), being short of breath and sweaty (moderate intensity), almost completely exhausted (high intensity)	
***Sedentary time***		
On a regular day, how many hours of sedentary time do you have (included work, school, and spare time)?	Reported in total hours/day (0–24)	Low sedentary time (reference) High sedentary time (≥8 h/day)
***Involvement in organized activities***		
How often do you participate in organized activity/volunteer work such as sports or political teams, religious communities, choirs, or others?	Never (0), rarely (0), 1–3 times/month (1), daily (2)	High participation in organized activities (reference) Low participation in organized activities (< 2 times/month)
How often do you participate in other activities such as clubs, meetings, meeting friends, exercise with friends/colleagues or others?		
***Social support***		
How many people are so close to you that you can count on them if you have serious personal problems?	None (1), 1–2 (2), 3–5 (3), ≥6 (4)	Strong social support (reference) Low of social support (< 12)
How much concern do people show in what you are doing?	No interest (1), little interest (2), neither great nor little interest (3), some interest (4), great interest (5)	
How easy is it to get practical help from neighbors if you should need it?	Very difficult (1), neither easy nor difficult (2), difficult (3), easy (4), very easy (5)	
***Sleep***		
To what degree have you experienced sleep problems this past week?	Not bothered (1), a little bothered (2), pretty much bothered (3), very much bothered (4)	No sleep problems (reference) Sleep problems (≥3)
***Perceived financial situation***		
If single-person household: Think about your total income. If you live together with others, think of the total income of everyone in the household. How easy or difficult is it for you to make ends meet daily with this income?	Very difficult (1), difficult (2), relatively difficult (3), relatively easy (4), easy (5), very easy (6), I don’t know (7)	Easy financial situation, ≤relatively easy and don’t know (reference) Difficult financial situation, (> 3)
***Age***		
Retrieved from the Central Population Register	≥18 years old	Continuous variable (low, reference)

Participants with mental distress were identified using the short version of the Hopkins Symptom Checklist (HSCL-5) which has proven to be a valid and reliable instrument developed from the original 25-item version to measure symptoms of anxiety and depression [27, 28]. Each of the five items used to measure mental distress were coded as follows: “not bothered” (coded 1), “a little bothered” (2), “somewhat bothered” (3), or “extremely bothered” (4). The total score of the HSCL-5 was calculated by adding the points from each item and divided by the number of questions. Participants who did not respond to all five HSCL-items were excluded from analyses (*n* = 427). Further, the variable was dichotomized, and a validated cut-off score of > 2.0 was used for identifying participants with mental distress [27].

Leisure-time physical activity level was assessed by asking three questions reflecting frequency, intensity, and duration. The data were processed as follows: frequency of physical activity was assessed by the question: *How often do you usually exercise? (Average)*. The response alternatives were coded: “Never” (coded 0), “less than once a week” (0.5), “once a week” [1], “2-3 times a week” (2.5), “4-5 times a week” (4.5), and “approximately every day” (6.5). Duration was assessed by the question: *For how long do you usually exercise each time? (Average)*. The response alternatives were coded: “Less than 15 minutes” (coded: 8), “15–29 min” [22], “30 minutes-1 hour” [29], and “more than 1 hour (75)”. Finally, intensity was assessed by the question: *How hard do you usually exercise? (Average)*. The response alternatives were: “Easy without being short of breath or sweaty”, “being short of breath and sweaty” or “almost completely exhausted”. Further, data reflecting frequency, duration and intensity of leisure-time physical activity were combined to identify individuals who achieved the WHO recommendations of at least 150 min of moderate-intensity aerobic physical activity or at least 75 min of vigorous-intensity aerobic physical activity per week [30].

Information about sedentary time was assessed by a single question identifying hours of sedentary time per day. A cut-off of ≥8 h were used based on results from another study showing that adults who spent less than 8 h per day sitting, were less depressed and anxious and had higher levels of vitality compared to those who spent more than 8 h per day sitting [31].

In the present study, involvement in organized activities was measured using two questions reflecting frequency of participation in different activities and the response alternatives for both items were coded as follows: “never” or “rarely” (coded: 0), “1-3 times per month” [1], “weekly” or “daily” [2] “. Total score for involvement in organized activities was achieved by adding both items which resulted in a score from 0 to 4 points. Further, the variable was dichotomized and < 2 points (i.e., < 2 times/month) was used to identify participants with low involvement (irregular participation) in organized activities.

The Oslo Social Support Scale (OSSS-3) is a brief and economic instrument to assess the level of social support and consists of three items that 1) reflect the number of close confidants, 2) the sense of concern from other people and the relationship with neighbors, and 3) the accessibility of practical help. Response alternatives for the first item were coded as follows: “No one” (coded: 1), “1–2” [2], “3–5” [3], and” 6 or more” [4]. For the second item, the response alternatives were coded: “no interest” (coded 1), “little interest” [2], “neither great nor little interest” [3], “some interest” [4], “great interest” [5]. Finally, response alternatives for the third item were coded: “very difficult” (coded:1), “difficult” [2], “neither easy nor difficult” [3], “easy” [4], and “very easy” [5]. The total score of the OSS-3 was calculated by adding the points from each item. In line with previous research [32], a score < 12 was used to identify participants without strong perceived social support in the present study, referred as low perceived social support.

Sleep problems were estimated by a single question focusing on subjective experience of having sleep problems the past week. The four response alternatives ranged from “not bothered” to “very much bothered”. Participants reporting “pretty much bothered” and “very much bothered” were considered having sleep problems in the present study.

Information about age and gender was obtained from The Central Population Register. Age was used as a continuous variable. Information about perceived financial situation, was used as a proxy for socioeconomic status. The response alternatives: “very difficult” and “difficult” were used to identify participants with perceived financial difficulties.

### Statistical analyses

Pearson chi-square tests were used to identify differences in financial situation, mental distress, leisure time physical activity, sedentary time, participation in organized activities, social support and sleep problems and independent sample t-test was used to analyse differences in age between men and women (Table 2). Pearson chi-square tests were also used to identify differences in mental distress between men and women with low leisure-time physical activity, high sedentary time, low participation in organized actives, low social support, and sleep problems (Tables 3 and 4). Further, crude multivariable logistic regression models and models adjusted for age and perceived financial situation were used to investigate possible associations between low level of leisure-time physical activity, high sedentary time, low involvement in organized activities, low social support, sleep problems and mental distress (Table 5). Separate models were presented for men and women. Pearson correlation tests revealed low pairwise correlations between independent variables in the models, indicating that multicollinearity was not present. Data-analyses were performed using IBM SPSS version 25, and the level of statistical significance was set to *p* < 0.05.

**Table 2:** Descriptive characteristics of male and female participants

	Men (*n* = 13,122)	Women (*n* = 14,925)	***P***-values
Age, mean (SD)	48.7 (16.4)	45.3 (16.0)	< 0.001
Hard financial situation, n (%)	2426 (19.4)	3121 (22.1)	< 0.001
Mental distress, n (%)	1539 (11.9)	2256 (15.3)	< 0.001
Low leisure-time physical activity, n (%)	8382 (64.3)	9584 (64.7)	0.550
High sedentary time, n (%)	4773 (37.5)	4861 (33.8)	< 0.001
Low participation in organized activities, n (%)	4256 (32.5)	3768 (25.3)	< 0.001
Low social support, n (%)	7645 (58.5)	7726 (52.0)	< 0.001
Sleep problems, n (%)	1648 (12.6)	2487 (16.7)	< 0.001

*Differences in categorical variables were analysed using chi-square tests. Difference in continuous variable (age) was

analysed using the independent sample t-test.

**Table 3:** Differences in mental distress between men and women with low level of leisure-time physical activity and high sedentary time

	Low leisure-time physical activity % (95% CI)			High sedentary time % (95% CI)		
	Men	Women	*P*-value*	Men	Women	*P*-value*
**Mental distress**	13 (12–14)	16 (16–17)	< 0.001	15 (13–16)	19 (18–20)	< 0.001
**No mental distress**	87 (86–88)	84 (83–84)		85 (84–87)	81 (80–82)	

* χ ^2^ tests were used to analyze differences in mental stress according to gender

**Table 4:** Differences in mental distress between men and women with low participation in organized activities, low social support and sleep problems

	Low participation in organized activities % (95% CI)			Low social support % (95% CI)			Sleep problems % (95% CI)		
	Men	Women	*P*-value*	Men	Women	*P*-value*	Men	Women	*P*-value*
**Mental distress**	17 (16–18)	24 (22–25)	< 0.001	17 (16–18)	23 (22–24)	< 0.001	45 (43–48)	44 (42–46)	0.461
**No mental distress**	83 (82–84)	76 (75–78)		83 (82–84)	77 (76–78)		55 (52–57)	56 (54–58)	

* χ ^2^ tests were used to analyze differences in mental stress according to gender

**Table 5:** Adjusted odds ratio (OR) and 95% confidence interval (CI) for mental distress in relation to low level of leisure-time physical activity, high sedentary time, low participation in organized activities, low social support and sleep problems among adults

		Model 1	Model 2	Model 3	Model 4
		Men	Men	Women	Women
		OR (95% CI)	OR (95% CI)	OR (95% CI)	OR (95% CI)
**Exposure variables**	**Leisure-time physical activity (PA)**				
	High PA	1 (ref)	1 (ref)	1 (ref)	1 (ref)
	Low PA	1.20 (1.05–1.37) **	1.18 (1.03–1.37) *	1.14 (1.02–1.28) *	1.05 (0.93–1.18)
	**Sedentary time**				
	Low sedentary time	1 (ref)	1 (ref)	1 (ref)	1 (ref)
	High sedentary time	1.36 (1.21–1.54) ***	1.32 (1.16–1.51) ***	1.38 (1.24–1.53) ***	1.25 (1.11–1.39) ***
	**Organized activities**				
	High involvement	1 (ref)	1 (ref)	1 (ref)	1 (ref)
	Low involvement	1.29 (1.14–1.46) ***	1.43 (1.25–1.64) ***	1.57 (1.41–1.75) ***	1.60 (1.42–1.80) ***
	**Social support**				
	Strong social support	1 (ref)	1 (ref)	1 (ref)	1 (ref)
	Low social support	2.82 (2.43–3.27) ***	2.55 (2.18–2.99) ***	2.98 (2.66–3.35) ***	2.71 (2.39–3.06) ***
	**Sleep problems**				
	No sleep problems	1 (ref)	1 (ref)	1 (ref)	1 (ref)
	Sleep problems	9.04 (7.96–10.27) ***	7.29 (6.35–8.37) ***	6.28 (5.65–6.99) ***	5.78 (5.15–6.50) ***
**Control variables**	**Age**^**a**^		0.97 (0.97–0.98) ***		0.96 (0.96–0.97) ***
	**Perceived financial situation**				
	Easy		1 (ref)		1 (ref)
	Hard		3.52 (3.09–4.03) ***		2.60 (2.32–2.91) ***

* *p* < 0.05, ** *p* < 0.01, ***p < 0.001

^a^ Age was modeled as a continuous variable and that the odds ratio is for a 1-year increment in age

## Results

The sample comprised 13.122 men (46.8%) and 14.925 women (53.2%) in which a total of 1538 men (12%) and 2256 women (15%) reported mental distress, respectively. Compared to women, a higher number of men reported high sedentary time, low participation in organized activities and low social support, whereas a higher number of women than men reported to have sleep problems (*p* < 0.001 for all). No gender differences in leisure-time physical activity were identified.

Among individuals with low leisure-time physical activity and high sedentary time, a higher proportion of women than men reported mental distress (16% vs. 13 and 19% vs. 15%, respectively). Likewise, among individuals with low participation in organized activities and low social support, a higher proportion of women than men reported mental distress (24% vs. 17 and 23% vs. 17%, respectively). Our results revealed no gender difference in the association between sleep problems and mental distress.

For men, binary logistic regression analyses adjusted for perceived financial situation and age showed that the odds of having mental distress was increased among individuals reporting low leisure-time physical activity (OR: 1.18; 95%CI: 1.03–1.37), high sedentary time (OR: 1.32, 1.16–1.51), low involvement in organized activities (1.43; 1.25–1.64), low social support (2.55; 2.18–2.99), and sleep problems (7.29; 6.35–8.37) compared to the rest of the male population.

For women, adjusted model showed that the odds of having mental distress was increased among those reporting high sedentary time (1.25; 1.11–1.39), low involvement in organized activities (1.60; 1.42–1.80), low social support (2.71; 2.39–3.06), and sleep problems (5.78; 5.15–6.50) compared to the rest of the female population. No significant association was shown between low leisure-time physical activity and mental distress among women after adjustment for age and financial situation.

## Discussion

Results from the present cross-sectional study showed an association between lifestyle risk factors and mental distress in a large sample of adults living in Southern Norway.

Our study revealed an association between low leisure-time physical activity and mental distress, but not for woman after controlling for possible confounders. A previous meta-analysis confirmed that physical activity seems to reduce depression by a medium effect and anxiety by a small effect in non-clinical populations [33] and results from a thirteen-year prospective cohort study indicated that both light and moderate to vigorous leisure-time physical activity may play a protective role against mental distress [34]. In line with our results, another meta-analysis has reported that gender may modify the effect of physical activity on incident depression [7], whereas another meta-analysis has suggested that the potential protective association of physical activity on depressive symptoms and depression is similar for men and woman [35]. The association between depression and physical activity appears to be bidirectional; physical activity may prevent and alleviate depressive symptoms in the general population and, in turn, having depressive symptoms in early adulthood may be perceived as a barrier to physical activity [36]. Results from previous studies suggest that regular physical activity recruits a process which results in enhanced emotional resilience to stress [37, 38]. However, possible mechanisms explaining observed effects of physical activity on mental distress are most likely complex and might be manifested at psychological (e.g., by improving mood, feelings of mastery, self-efficacy) and neurophysiological (e.g., by increasing synthesis and release of neurotransmitters and neurotrophic factors associated with neurogenesis, angiogenesis and neuroplasticity) levels [39, 40].

Further, our results confirmed an association between high sedentary time (≥8 h/day) and mental distress in both men and women. Systematics reviews have suggested a small positive association between sedentary time and symptoms of both anxiety and depression, but also highlighted that only a few studies, with partly inconsistent results, had examined this relationship [41, 42]. In addition, previous cross-sectional studies have confirmed an association between high sedentary time and mental distress [29, 43]. A possible explanation for the observed relationship between sedentary time and mental distress is that high sedentary time may displace physical activity, which has been shown to reduce levels of mental health problems [44]. High sedentary time may also be an independent risk factor of mental distress as uninterrupted sedentary time seems to moderate and deleterious insulin sensitivity changes, glucose tolerance, and plasma triglyceride levels, which indirectly could affect mood and well-being [45].

Research has indicated that participation in organized activities provide a sense of belonging, value, and attachment [24], and may thereby have a positive influence on mental health. Whereas our study used a broad approach to identify participation in organized activities by including focus on both involvement in physical activity groups, political teams, volunteer work, religious communities, as well as clubs, most previous studies have focused solely on whether participation in physical activity groups may be associated with improved mental health. Results from the present study indicated that low involvement in organized activities was associated with increased odds of mental distress, both among men and women. These findings are supported by results from prospective studies among European adults suggesting that involvement in social organized activities predicted higher quality of life and lower level of depressive symptoms over time and be a protective factor against the onset or development of chronic conditions, especially among individuals with few close social ties [16, 46]. Finally, a Norwegian population-based study has suggested a dose-response effect of participation in organized activities on perceived health, anxiety, depression, and satisfaction with life in both women and men, and that men who engaged specifically in receptive, rather than creative, cultural activities reported better health-related outcomes [47]. However, few longitudinal and experimental studies have examined gender dependent effects of participation in different organized activities on mental health.

Similar to our results, several other studies have also reported that lower level of perceived social support are associated with mental distress in both men and women [48, 49]. Furthermore, among older individuals who lacked social support, women seemed to need more emotional support whereas men seemed to need more tangible support [48]. A longitudinal study among British adults has also confirmed a bidirectional relationship between social support and mental health and that the relationship varied over the life course [50]. Furthermore, low social integration among peers in adolescence has recently been shown to predict higher levels of depression among US adults [51], and systematic reviews have reported evidence linking low of social support, social isolation, and loneliness to mental health problems [25, 52]. On the other hand, having trust in people, feeling safe in the community, and having social reciprocity has been identified as factors associated with lower risk of mental distress [53]. Previous studies have argued that high perceived social support could play a key role in reducing risk factors of mental distress caused by negative life events, such as job loss and marriage break-down [54, 55].

Finally, the current study showed that having sleep problems was strongly associated with increased odds of mental distress in both men and women. Results from previous studies indicated a U-shaped association between sleep duration and mental distress [56], and that individuals who experienced both insomnia and short (< 7 h) sleep duration had increased risk of chronic mental health symptoms [57]. Moreover, a study among Norwegian adults confirmed that different symptoms of insomnia seemed to play a key role in rising of anxiety levels [58] and a meta-analysis indicated that non-depressed subjects with insomnia had a twofold risk of developing depression compared to those with no sleep difficulties [59]. Studies have also suggested a bidirectional relationship between sleep problems and mental distress, and that mental distress increase the risk of experiencing insomnia later in life [60, 61]. Underlying psychophysiological mechanisms through which insomnia, or general sleep problems, predicts mental distress are still not clear. Previous studies, however, have raised attention to the role of sleep in emotion regulation [62], and from a neurobiological perspective, a dysfunction in sleep-wake regulating neural circuits may lead to alterations in emotional reactivity [63].

Several methodological limitations of the present study should be considered. This study’s main limitation is the cross-sectional design which precludes inferences about causality. Further, the present study relied on self-reported measures which are prone to memory and recall bias, as well as social-desirability bias. Despite a limited participation rate and a higher proportion of high educated participants compared to the total adult population in Norway (48% versus 34%) [64], a major strength of the present study was the large sample drawn randomly from a general population. Another strength is that we used validated tools to measure mental distress (HSCL-5) and social support (OSS-3) [65–67], and that data reflecting frequency, duration and intensity and duration of leisure-time physical activity were combined to identify individuals who achieved the WHO recommendations [30]. Further, perceived financial difficulties, has previously been identified as a major contributor to mental distress [68] and was therefore used as a measure of socioeconomic status in the present study.

## Conclusion

The present comprehensive study among Norwegian adults from the general population confirmed increased odds of mental distress among those with high sedentary time, low participation in organized activities, low social support and sleep problems. However, future longitudinal studies are needed to confirm a possible causal relationship between different lifestyle behaviors, participation in organized activities and social support, and mental distress, and thereby contribute further to developing targeted strategies for prevention of mental distress and illness in society.

## Data Availability

The Norwegian Institute of Public Health (NIPH) is legally responsible and owner of the Norwegian Counties Public Health Survey. Data may be provided from NIPH on reasonable request.
